# Reconstructing human population history from dental phenotypes

**DOI:** 10.1038/s41598-017-12621-y

**Published:** 2017-10-02

**Authors:** Hannes Rathmann, Hugo Reyes-Centeno, Silvia Ghirotto, Nicole Creanza, Tsunehiko Hanihara, Katerina Harvati

**Affiliations:** 1 Paleoanthropology, Senckenberg Centre for Human Evolution and Palaeoenvironment, Eberhard Karls University of Tübingen, Tübingen, Baden-Württemberg 72070 Germany; 2 DFG Center for Advanced Studies ‘Words, Bones, Genes, Tools’, Eberhard Karls University of Tübingen, Tübingen, Baden-Württemberg 72070 Germany; 3 Department of Life Sciences and Biotechnologies, University of Ferrara, Ferrara, Emilia-Romagna 44121 Italy; 4 Department of Biological Sciences, Vanderbilt University, Nashville, Tennessee 37212 United States of America; 5 Department of Anatomy, Kitasato University School of Medicine, 1-15-1 Kitasato, Minami-ku, Sagamihara 252-0374 Japan

## Abstract

Dental phenotypic data are often used to reconstruct biological relatedness among past human groups. Teeth are an important data source because they are generally well preserved in the archaeological and fossil record, even when associated skeletal and DNA preservation is poor. Furthermore, tooth form is considered to be highly heritable and selectively neutral; thus, teeth are assumed to be an excellent proxy for neutral genetic data when none are available. However, to our knowledge, no study to date has systematically tested the assumption of genetic neutrality of dental morphological features on a global scale. Therefore, for the first time, this study quantifies the correlation of biological affinities between worldwide modern human populations, derived independently from dental phenotypes and neutral genetic markers. We show that population relationship measures based on dental morphology are significantly correlated with those based on neutral genetic data (on average *r* = 0.574, *p* < 0.001). This relatively strong correlation validates tooth form as a proxy for neutral genomic markers. Nonetheless, we suggest caution in reconstructions of population affinities based on dental data alone because only part of the dental morphological variation among populations can be explained in terms of neutral genetic differences.

## Introduction

In archaeological and paleontological studies, dental phenotypic data are often used to estimate biological relatedness among past human groups, in order to reconstruct migration events, population histories, or hominin phylogenies^1–11^. Dental morphology has become a favored dataset primarily because teeth are generally well preserved in the archaeological and fossil record, even when associated skeletal and DNA preservation is relatively poor. Their better state of preservation results in teeth being recovered in higher quantities and, therefore, allows studies to employ larger samples and more robust statistical analyses. Furthermore, tooth form has been proposed to be highly heritable, selectively neutral, and evolutionarily conservative, thus, providing an excellent proxy for neutral genetic data^12,13^. Tooth crowns develop relatively early in the life of an individual and their form is not altered after full formation, except by wear or pathology. Finally, dental phenotypic data can be sampled in a non-destructive, cost-efficient, and straightforward manner using crown width and length measurements (hereafter, dental metrics) or visual scoring of well-established crown and root shape variants (hereafter, dental non-metric traits).

Despite the popularity of population genetic studies utilizing dental phenotypes as proxies for genetic markers, less than a handful of studies have attempted to directly test the level of congruence between population distance measures based on these two data types^14–17^. Those previous investigations found contradicting results, with some of them reporting weak to strong correlations, whereas others found that dental and genetic distances produced fundamentally different patterns of group relationships. Thus, the utility of dental morphology as an efficient proxy for genetic data, formally tested in human population genetic analyses, is currently unresolved. It also has to be noted that those previous studies were limited by several factors. First, most used serological data as genetic markers; however, contemporary genetic studies commonly utilize either single nucleotide polymorphisms (SNPs) or short tandem repeats (STRs) due to their highly polymorphic nature^18^. In fact, it has been proposed that phenotypic variation should be compared to both neutral genomic data types^19–21^ since the mutational rate of sequence change and the apportionment of modern human genetic variation is different in SNPs and STRs^22^. Second, most previous studies were limited to dental non-metric trait data; however, dental metrics are another important data source for biological distance studies and some researchers argue that crown measurements may be collected with lower observer error than dental non-metric traits^23^. Third, all previous studies were limited to regional scales, with some of them analyzing only a few population samples, which reduces the power of statistical correlation tests between dental and genetic distance estimates. A study seeking to investigate dental morphological and neutral genetic correspondence with a large set of globally distributed population samples is still pending.

**Figure 1:**
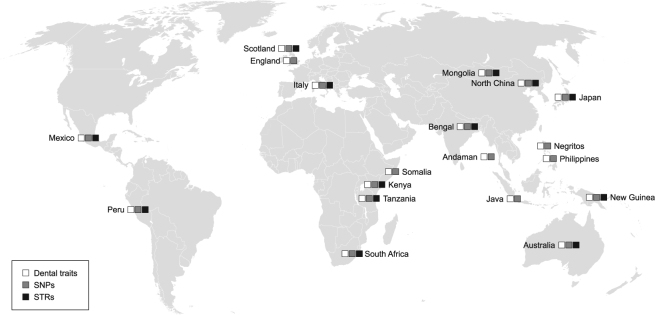
Location of the modern human population samples used in this study. White squares indicate that the population was sampled for dental metric and non-metric traits. Grey squares indicate that the population was sampled for single nucleotide polymorphisms (SNPs). Black squares indicate that the population was sampled for single tandem repeats (STRs). Word map modified from BlankMap-World6, available at https://commons.wikimedia.org/wiki/File:BlankMap-World6.svg (Public Domain).

Here, for the first time, we seek to test for correlations of biological affinities among globally distributed modern human populations, derived independently from diverse dental phenotypic markers (metrics and non-metric traits) and neutral genetic loci (SNPs and STRs). To do so, we first matched genomic and dental phenotypic population samples from around the world using existing databases. Matched SNP and dental phenotypic data were available for 19 populations and matched STR and dental phenotypic data were available for a subset of 13 populations (Fig. 1). We then used the R-matrix method^24^ to calculate pairwise population kinship coefficients (*r*_ij_) utilizing the genomic and dental phenotypic datasets independently. R-matrix analyses, founded on population and quantitative genetic theory, are most useful for comparing patterns of biological similarity from different types of data and, additionally, allowed us to correct for the confounding effects of genetic drift in different regions of the world by including estimates of effective population sizes (*N*_e_)^19,24,25^. Finally, we statistically assessed the associations between genomic and dental phenotypic kinship estimates using Mantel correlation tests. Dow-Cheverud tests were then used to determine whether dental metrics or dental non-metric traits are better suited to track neutral genomic relationships as calculated from SNP and STR data.

## Results

**Figure 2:**
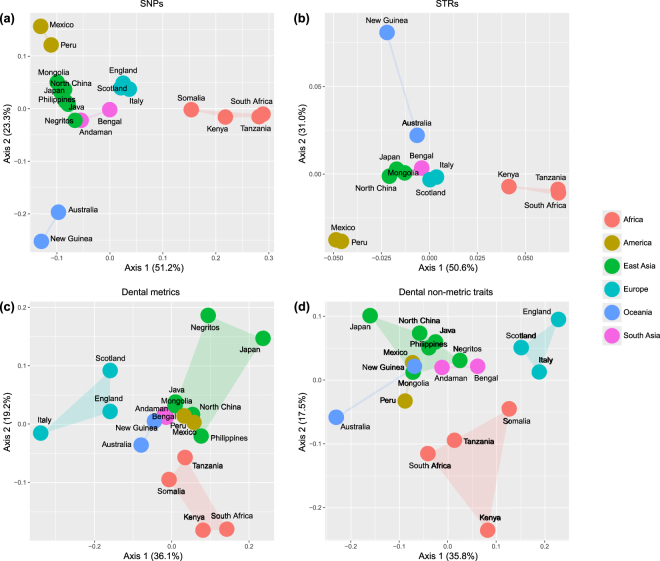
Biological distances among human populations (*d*_ij_) generated from neutral genetic and dental phenotypic data. Figures show scatterplot of the first two principal coordinates of: (**a**) *d*_ij_ distances generated from SNPs; (**b**) *d*_ij_ distances generated from STRs; (**c**) *d*_ij_ distances generated from dental metrics; and (**d**) *d*_ij_ distances generated from dental non-metric traits.

Figure 2 illustrates biological affinities among globally distributed modern human populations, derived independently from dental phenotypic markers and neutral genetic loci. While SNPs and STRs gave largely concordant results, dental metric and non-metric traits revealed a somewhat different pattern. Overall, dental phenotypes successfully classified populations in broader geographic and continental areas.

**Figure 3:**
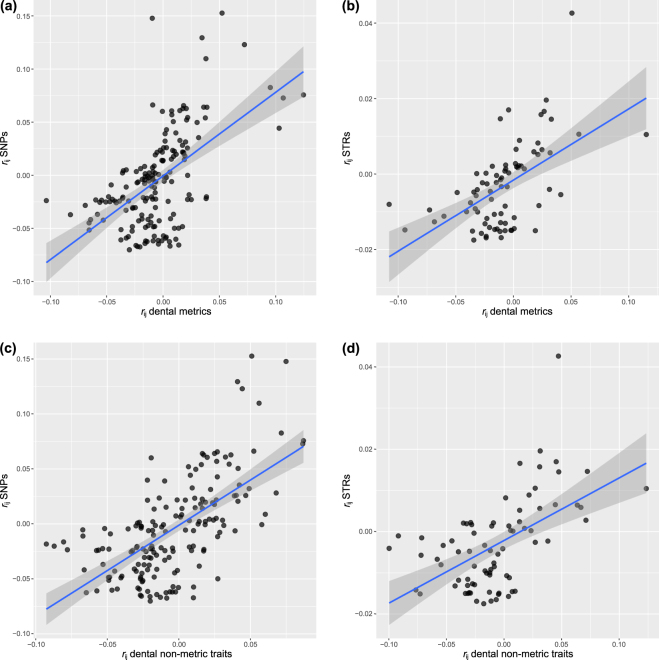
Regression of pairwise kinship coefficients among human populations (*r*_ij_) generated from neutral genetic and dental phenotypic data. Figures show scatterplot, linear regression line, and 95% confidence interval of: (**a**) *r*_ij_ values generated from SNPs versus *r*_ij_ values generated from dental metrics; (**b**) *r*_ij_ values generated from STRs versus *r*_ij_ values generated from dental metrics; (**c**) *r*_ij_ values generated from SNPs versus *r*_ij_ values generated from dental non-metric traits; and (**d**) *r*_ij_ values generated from STRs versus *r*_ij_ values generated from dental non-metric traits.

Our results show that kinship estimates between human populations based on dental phenotypes are significantly correlated with those based on neutral genetic data (Table 1, Fig. 3). The correlation values were relatively strong and similar for all four data type comparisons. Dental metric variation explained approximately 31% of the neutral genetic differences among populations as calculated from SNPs and STRs. Dental non-metric variation explained about 40% and 30% of the neutral genetic differences among populations as calculated from SNPs and STRs, respectively.

**Table 1:** Mantel and Dow-Cheverud tests.

	SNPs (19 populations)	STRs (13 populations)
Dental metrics	0.558 (<0.001)^1^	0.556 (<0.001)^1^
Dental non-metric traits	0.635 (<0.001)^1^	0.547 (<0.001)^1^
Dental metrics vs. Dental non-metric traits	0.074 (0.155)^2^	−0.008 (0.464)^2^

^1^Mantel test of dental phenotypic kinship coefficients (*r*_ij_) against neutral genetic *r*_ij_. Reported values are Pearson correlation coefficients (*r*) and two-tailed significance (*p*, in parentheses) after 10,000 permutations. All comparisons are statistically significant after Bonferroni correction for multiple testing at α = 0.025.

^2^Dow-Cheverud test of dental metric *r*_ij_ versus dental non-metric *r*_ij_. Reported values are correlation coefficients (*p*1Z) and two-tailed significance (*p*, in parentheses) after 10,000 permutations. Positive correlation values indicate that dental metrics are more strongly correlated with neutral genetics. Negative correlation values indicate that dental non-metric traits are more strongly correlated with neutral genetics. None of the results are statistically significant at α = 0.05.

Table 1 also presents the results of the Dow-Cheverud test, which determined whether dental metrics or dental non-metric traits are significantly more strongly correlated with either SNP or STR markers. None of the comparisons were significant, indicating that dental metrics and dental non-metric trait data are both comparably well-suited in tracking neutral genetic relationships as calculated from SNPs and STRs.

## Discussion

Our results validate the use of dental phenotypic data to infer neutral genetic relationships among human populations. This, at least to some extent, confirms the previous hypothesis^13^ that the worldwide human dental variation was primarily generated by random processes of genetic drift. We also found that different dental phenotypic data types, i.e. metric and non-metric traits, are both well-suited in serving as proxies for neutral genetic markers. This result supports the previous finding that different dental phenotypic data types give concordant but varied results, and the conclusion that reconstructions of population history are best served when both lines of evidence are investigated^23^. Although all correlations between dental phenotypes and neutral genetic markers were highly significant, their correlation coefficients indicated that only part of the dental phenotypic variation can be explained in terms of neutral genetic differences. Other non-stochastic factors therefore account for a large portion of the variation in dental morphology of modern humans. Because we controlled for the effect that sexual dimorphism, pathology, and wear can have on teeth, we reason that a substantial portion of the variation can be explained by natural selection on dental morphology. This interpretation is consistent with previous inferences and direct genomic evidence linking non-neutral gene variants with specific tooth characteristics^26–28^.

While it has been shown or suggested that linguistic and skeletal phenotypic variation can correlate differentially with genomic variation based either on SNPs or STRs^19,29^, our results do not suggest this to be the case for dental phenotypic variation. Despite the differences in the mutational change and evolution of SNPs and STRs^30,31^, both genomic datasets showed the same pattern and a similar degree of correlation with dental phenotypic variation.

The level of agreement between kinship estimates based on dental non-metric traits and STRs found in this study is comparable to that previously found in the only other study that has tested the association of dental morphology and neutral genomic variation^17^. That study compared dental and genetic distances among four modern groups in Kenya using paired data from 295 individuals. They calculated dental distances using a Mahalanobis-type (D^2^) distance for binary data^32^ derived from nine non-metric crown traits. Genetic distances were estimated using a delta-mu squared (Ddm) distance^33^ utilizing 42 STR loci. They compared both distance matrices with a Mantel test and found a moderate to strong positive correlation between the two distance types, although this result was not significant (*r* = 0.500, *p* = 0.21). The correlation coefficient reported here is slightly higher (*r* = 0.547, *p* < 0.001). This could be due to the larger battery of dental traits employed (12 traits vs. 9 traits), the higher geographic scale of analysis (global scale vs. regional scale), and/or the use of different biological relationship measures (R-matrix comparisons vs. D^2^ against Ddm). Moreover, the correlation reported here is highly significant, whereas the correlation presented by ref.^17^ was not, albeit that result was probably due to the Mantel test design based on only four populations.

More broadly, the quantified degree of correspondence between dental and neutral genetic variation reported here is similar to that found for other skeletal cranial elements^19,34–40^. Dental and cranial phenotypes are therefore equally well-suited for reconstructing genetic relationships among populations. However, we caution that previous studies on the association of cranial and genomic variation are not directly comparable to ours since different populations have been sampled and diverse methodological approaches have been employed.

It is important to point out that our study is biased toward not finding significant correlations between variation in neutral genetics and dental phenotypes. First, we compared matched but unpaired datasets, such that dental samples were from different individuals than those sampled for SNP and STR sequencing. Although it is a common and practical procedure to compare unpaired data at a global scale^19,34–40^, it is likely that it results in sampling bias given that genetic variation between human populations is low compared to within-population variation^41^. Second, it is possible that the dental metric and non-metric datasets employed in this study do not capture adequate phenotypic variation. Our metric dental dataset comprises well-established crown width and length measurements, but could be complemented with alternative measures, such as diagonal crown measurements and cervical diameters at the cement-enamel junction^42^, or other measurements that derive from innovative and more robust 3D imaging techniques not requiring the use of hand-held calipers. Likewise, our dental non-metric dataset was limited to 12 traits while more than 30 traits have been identified as useful in detecting population relationships^43^. Furthermore, we utilized binary non-metric dental trait counts, although recent research has shown that dichotomization of ordinal-scaled data into simplified binary categories may skew biological distance results^44,45^. Given the limitations of our study, the levels of association between neutral genetic and dental phenotypic kinship estimates reported here must therefore be considered as minimum values and not as exact correlations. Paired data from individuals sampled worldwide, as has been employed at a smaller scale^17^, would provide a more accurate estimate of genetic and dental phenotype associations.

In conclusion, our results confirm that dental phenotypic data can be used as a proxy for neutral genomic data in studies of population relatedness, although we suggest caution and careful choice of dental features because only part of the dental variation among populations can be explained in terms of neutral genetic differences. Future work should focus on (1) analyzing paired neural genetic and dental phenotypic datasets from the same individuals, (2) using globally distributed population samples, (3) collecting both conventional and alternative dental metric and non-metric traits, and (4) comparing patterns of biological similarity from genetic and dental phenotypic data using the same quantitative genetic model. By performing several comparisons using different dental fields and different combinations of dental metric and non-metric traits, future work could potentially identify dental data combinations that are most useful for tracking human population history.

## Materials and Methods

### Matching population samples

Materials for this study comprise four different types of data: SNP allele frequencies, STR allele frequencies, dental metrics, and dental non-metric traits. All data were taken from existing databases. We matched datasets for several globally distributed modern human populations for which both genetic and phenotypic data were available (Fig. 1, Supplementary Table S1). Populations were chosen for inclusion in this study based on two criteria: first, availability of sufficient number of dental phenotypic specimens (i.e. both dental metrics and non-metric traits); and, second, availability of neutral genetic data (i.e. SNPs and/or STRs). In instances where exact population matches could not be achieved, a geographically similar population with ethno-linguistic affinities was selected. Matched SNP and dental phenotypic data were available for 19 populations; however, STR data were only available for a subset of 13 populations. We note that the matched populations are unpaired samples; that is, dental samples derive from different individuals than the genetic samples.

### Neutral genetic data

SNP allele frequencies were collated from various datasets^46–55^ for 19 populations comprising n = 1652 individuals sharing 1778 markers. The SNP data were merged using the plink 1.07 software^56^ and polymorphisms possibly causing strand ambiguities (A/T or C/G) were removed. We then exploited the extent of linkage disequilibrium (LD) observed in each population to obtain an estimate of the effective population size (*N*_e_) through time. Linkage disequilibrium levels have been estimated independently in each population using all SNP markers available for that population. We evaluated for each SNP the genetic map position, and for each pair of SNPs separated by less than 0.25 cM we quantified LD as the r^2^_LD_, calculated in plink 1.07. All observed r^2^_LD_ values were then binned into one of 250 overlapping recombination distance classes, from 0.005 cM to 0.25 cM. Following refs^25^,^57^, pairs of SNPs separated by less than 0.005 cM were not considered, and the adjusted r^2^_LD_ values were corrected for sample size. We finally calculated the effective population size in each recombination distance class through the formula: *N*_e_ = (1/4c)[1/r^2^_LD_ − 2], which corresponds to the effective population size 1/2c generations ago, where c is the distance between loci, expressed in Morgans^58^. The long-term *N*_e_ for each population was then calculated as the harmonic mean of the values of N_e_ over all the recombination distance classes. The estimated *N*_e_ values for each population are reported in the Supplementary Table S1.

In addition to the SNP data, we analyzed a dataset of STR allele frequencies that combined data from several studies; the merging of data is described in ref.^59^. Specifically, we used their MS5255 dataset, which has genotype data from 645 loci for 265 worldwide populations. At each locus, allele sizes are recorded for each individual. Following refs^60^,^61^, we tested for individual outliers by generating a matrix of individuals by alleles, performing a principal components analysis on this matrix, and defining an outlier as an individual with a score more than six standard deviations from the mean of any of the first four principal components. None of the individuals met these criteria, so all individuals were considered for further population-level analyses. We then restricted the dataset to n = 265 individuals in the 13 populations with both STR and dental data as described above.

### Dental phenotypic data

The dental phenotypic data were collected by one of us (T.H.) and comprise dental metrics and dental non-metric traits from mostly the same individuals. Several samples are from collections of known age and sex. When demographic data were not available, age and sex were determined by T.H. using standard osteological methods^62^. When possible, approximately equal numbers of adult males and females were measured for both dental datasets for each population. However, we note that overall, the datasets are biased in representing more males. Detailed information on the composition of the morphological datasets, such as country of origin, ethnic affiliation, and cultural background is given elsewhere^63,64^. We excluded samples older than 2.000 years in order to avoid temporal bias.

The dental metric dataset consists of mesio-distal and bucco-lingual crown diameters of all teeth recorded for each individual (up to a total of 28 metric variables, excluding third molars). Only right teeth were measured, but when a right tooth was missing, damaged, or affected by wear or pathology, the corresponding left antimere was measured. All measurements were recorded according to the procedures of ref.^12^ using a digital sliding caliper accurate to 0.01 mm. T.H. quantified his level of intra-observer error by separately re-measuring a Japanese sample; measuring error was found to be insignificant^63^. Because not every tooth could be observed for each individual due to poor preservation or pathology, the dataset comprises large amounts of missing values. The multivariate statistical methods performed here require complete datasets; however, removing individuals with missing values would eliminate the bulk of the sample. Thus, missing data were imputed following ref.^65^ using the *k*-nearest neighbor (kNN) algorithm, conducted in the software R 3.3.1^66^ using the VIM package^67^. The kNN algorithm searches the entire dataset for cases most similar to the one with missing data and generates a mean to replace the missing value(s). Prior to imputation, individuals with more than half of the measurements missing were removed from the analysis. In this way we ensured that less than 22% of the final dataset requires imputation (down from 56%). Raw measurements were then converted into shape variables by dividing each measurement by the geometric mean for all the measurements in each individual^68^. This standardization procedure removes gross size from the data in order to assess differences in the proportionate contribution of individual variables to overall tooth size. This procedure also has the advantage to adjust for size differences between individuals that may result from sexual dimorphism. A table listing the summary statistics of the dental metric dataset is provided in the Supplementary information (Table S2).

The dental non-metric trait dataset consists of observations for 15 morphological variables in the permanent dentition according to procedures detailed in ref.^64^. The 15 traits include characteristics attributed to the Asian^69^, European^70^, and sub-saharan African dental complex^71,72^, as well as the key crown traits that distinguish continental Southeast Asians from island Southeast Asians^73^. Most (14 of 15) traits follow the widely used Arizona State University Dental Anthropology System (ASUDAS) described by ref.^43^. This system has as reference set of dental casts illustrating expression levels for various traits and specific instructions that ensure a standardized scoring procedure that minimizes observer error. Although observations were made on both antimeres, scoring followed the individual count method^74^, where a trait was counted only once per dentition, regardless of whether or not the trait appeared bilaterally. In cases where a trait was expressed asymmetrically, we followed the standard ASUDAS protocol and scored the side with the highest expression level^4,5,75,76^. The dental observations were originally scored in a graded fashion and were subsequently dichotomized into simplified categories of presence or absence following the dichotomization thresholds detailed in ref.^64^. Thus, our final dataset consists of binary dental trait information (i.e. 0 = absent, 1 = present) for each individual. The multivariate statistical methods performed here can handle incomplete datasets; however, the amount of missing data should be reduced as much as possible in order to prevent non-positive-semidefinite dispersion matrices^44^. We therefore removed the most incomplete variables and individuals from the analysis in a systematical stepwise manner so that the final dataset consists of less than 40% missing data (down from 60%). Most dental traits listed in the ASUDAS have low or no sexual dimorphism^13^, which allows for pooling of sexes^4,64,76^. A table listing the final dental non-metric dataset is given in the Supplementary information (Table S3).

### Generating population affinity matrices

We independently estimated genetic and dental phenotypic affinities between the sampled populations using the R-matrix method. The R-matrix method was originally developed to work with allele frequency data^77^ and was later modified for use with morphometrics^24^ and non-metric traits^78^. These extensions make R-matrix analyses most useful for comparing patterns of biological relationships from different types of data^79^. The off-diagonal elements of an R-matrix quantify the biological relationship between population pairs with values ranging from +1 to −1. Those values are covariances about the regional centroid and are defined as average kinship coefficients (*r*_ij_). Positive *r*_ij_ values indicate that two populations exhibit greater biological similarity than on average, and negative *r*_ij_ values denote that two populations are more distinct than on average. Moreover, the R-matrix can be scaled by weighing the samples by their population sizes in order to account for the confounding effects of genetic drift on small populations. Here, we included point values of effective population size (*N*_*e*_) derived from levels of genetic linkage disequilibrium (values are reported in the Supplementary Table S1). The phenotypic R-matrices were calculated with a heritability estimate of *h* ^2^ = 0.5, reflecting the approximate average of various heritability estimates of dental anatomy based on twin and family studies^63,64^.

Genetic R-matrices were generated from the allele frequency data using the RMAT 1.2 software, following the model described by ref.^77^. Genetic R-matrices were constructed for all 19 populations using the SNP data and for the subset of 13 populations using the STR data. Dental metric R-matrices were generated from the crown width and length measurements using the RMET 5.0 software, following the model described by ref.^24^. We constructed two dental metric R-matrices; one for the 19-population setup and a second for the 13-population subset. Dental non-metric R-matrices were generated from the discrete crown traits in R 3.3.1, following the methodology detailed in refs^78,80^,^81^. As with the dental metric dataset, we constructed two dental non-metric R-matrices; one for the 19-population dataset and a second for the 13-population subset. All estimated R-matrices are reported in the Supplementary information (Tables S4–S9).

### Comparing population affinity matrices

To measure the degree of association between genetic and dental phenotypic population kinship coefficients, we followed the protocol set forth by refs^35^,^40^ and compared the off-diagonal R-matrix values using Mantel tests. Mantel tests measure the congruence between two matrices against a null model and assess statistical significance via a permutation procedure^82^. Genetic R-matrices based on SNPs and STRs were compared independently against the phenotypic R-matrices based on dental metrics and dental non-metric traits. The Mantel tests were conducted in R 3.3.1 using the vegan package^83^. Correlation significance was determined after 10.000 random permutations and significance levels were set to α = 0.025 to correct for multiple comparisons (Bonferroni correction: α = 0.05/2). We interpreted correlation strength following the convention of ref.^84^. We furthermore visualized the association of R-matrix values in regression plots, generated in R 3.3.1 using the ggplot2 package^85^. In addition to the Mantel tests, we performed Dow-Cheverud tests^86^ in order to determine whether dental metrics or dental non-metric traits could be considered significantly more strongly correlated with neural genetic variation as calculated from SNPs and STRs. Dow-Cheverud tests were conducted in R 3.3.1. Correlation significance was determined after 10.000 random permutations and significance levels were set to α = 0.05. We furthermore visualized population affinities generated from neutral genetic and dental phenotypic data by deriving pairwise distances from the R-matrices^24^ and plotting them using principal coordinates analysis in R 3.3.1 employing the vegan^83^ and ggplot2^85^ packages.

### Data availability

The data that support the findings of this study are available from T.H., S.G., and N.C. but restrictions apply to the availability of these data, which were used under license for the current study, and so are not publicly available. Data are however available from the authors upon reasonable request and with permission of T.H., S.G., and N.C.

## Electronic supplementary material

Supplementary Information
