# Supplementary material for: Reconstructing human population history from dental phenotypes
Source: Sci Rep. 2017 Oct 2;7:12495. doi: 10.1038/s41598-017-12621-y (PMC5624867; doi:10.1038/s41598-017-12621-y)
Supplement: Supplementary file 1 — Supplementary Information [file 41598_2017_12621_MOESM1_ESM.pdf]

Supplementary Information

Reconstructing human population history from dental phenotypes

Hannes Rathmann, Hugo Reyes-Centeno, Silvia Ghirotto, Nicole Creanza, Tsunehiko Hanihara, Katerina Harvati

Contents

Supplementary Table S1. Matched population samples, sample sizes (n), and estimates of effective population sizes (N_e) ... 2

Supplementary Table S2. Mean ($\bar{x}$) and standard deviation (SD) of the dental metric dataset after the removal of individuals with high amounts of missing values, the imputation of missing data, and the transformation into shape variables ... 3

Supplementary Table S3. Frequencies of positive observations (%) and number of individuals scored (n) of the dental non-metric dataset after the removal of individuals and variables with high amounts of missing values ... 4

Supplementary Table S4. Kinship coefficients (r_{ij}) among 19 population samples based on SNPs ... 4

Supplementary Table S5. Kinship coefficients (r_{ij}) among 19 population samples based on dental metrics ... 5

Supplementary Table S6. Kinship coefficients (r_{ij}) among 19 population samples based on dental non-metric traits ... 5

Supplementary Table S7. Kinship coefficients (r_{ij}) among 13 population samples based on STRs ... 6

Supplementary Table S8. Kinship coefficients (r_{ij}) among 13 population samples based on dental metrics ... 6

Supplementary Table S9. Kinship coefficients (r_{ij}) among 13 population samples based on dental non-metric traits ... 6

References ... 7

Supplementary Table S1. Matched population samples, sample sizes (n), and estimates of effective population sizes (N_e)

Population	Dental phenotypic samples	Dental metrics[1]		Dental nonmetric traits[2]		SNP samples	STR samples[3]	N_e estimate
		Male (n)	Female (n)	Male (n)	Female (n)	Sample (n)	Sample (n)	
AND Andaman	Recent Andamanese	25	14	31	21	Great Andamanese (7)[4], Onge (9)[4]	–	2,881
AUS Australia	Recent Australians from Queensland, Southern Australia, Western Australia and New South Wales	77	16	66	34	Australians (12)[5], Australians WGA (7)[6]	Australian (10)	4,205
BEN Bengal	Recent inhabitants from West Bengal	32	11	60	36	Bengali (16)[7]	Bengali (27)	3,839
ENG England	Mid-Victorian, Pre-17th century cemetery at Spitalfields, Eastern region of London	67	45	79	46	United_Kingdom_POPRES (751)[8]	–	5,946
ITA Italy	Recent inhabitants from Southern Italy and Sicily	34	0	81	28	Italy_POPRES (255)[8]	Tuscan (8)	7,167
JAP Japan	Recent Japanese from Tokyo and Miyako Island	88	37	37	34	Ryukyuan (49)[7], JPT(Japanese_in_Tokio) (91)[9]	Japanese (29)	5,366
JAV Java	Recent Javanese mainly from Jakarta	57	6	67	20	Javanese (34)[7], Japanese (19)[7]	–	5,800
KEN Kenya	Recent inhabitants of Kikuyu, Nairobi, Teita Hills	26	5	62	40	Kikuyu (4)[10], Luhya_Kenya_LWK (8)[10], Luo (9)[10], Masai_Ayodo (3)[10], Masai_Kinyawa_MKK (10)[10]	Kikuyu (21)	10,933
MEX Mexico	Recent inhabitants of Sierra Madre of Durargo, Tombat Xico, Jarasco, Tarasco, Yucatan, Duraugo, Pueblo, Chihuahua, Tarahumare, Papago; Cora, Oroyo del Santa Rosa, Nixtalpa, Territory	25	6	62	39	Totonac (24)[11], Zapotec (10)[10], Mixtec (10)[10], Mayan (21)[10]	Maya (25)	2,808
MON Mongolia	Recent Mongolians from Ulaanbaatar	46	32	87	81	Mongola (11)[10]	Mongola (10)	3,600
NCH North China	Recent Northeastern Chinese from Heilongjiang and Jirin Provinces	36	3	49	4	Daur (9)[10], Hezhen (9)[10], Oroqen (9)[10]	Daur (10), Hezhen (9), Oroqen (10)	5,832
NEG 'Negritos'	Recent Philippine 'Negritos'; Aeta and Agta from Luzon	30	8	35	13	Agta (8)[7], Ati (23)[7], Ayta (8)[7], Iraya (9)[7], Mamanwa (19)[7], Mamanwa (11)[5], Manobo (16)[5]	–	4,427
NGU New Guinea	Recent inhabitants from Purari River Delta, Fly River Delta, Sepik River Delta, etc.	20	11	101	105	Papuan (18)[10], New Guinea (20)[6]	Sepik (20)	3,436
PER Peru	Recent inhabitants from Cerro del Oro, Cajamarquilla, San Damian, Chilca,Coyugo, Cinco Cerros, Huacho; Casa Grande, Macato, Huaras Lupo, Masca	29	13	75	51	Quechua_Coriell (5)[10]	Quechua (20)	2,700
PHI Philippines	Recent Filipinos, Tagalog, Bilan, Bisaya, Igorot, Ifugao and other tribes; mainly from Luzon and Mindanao Islands	30	11	66	43	Filipino (20)[7], Filipino (20)[7], Filipino (19)[7]	–	5,672
SAF South Africa	Recent Zulu from Pietremanitzburg, Mantatee, Tulu, Tambuki, Natal, South Africa	35	0	54	3	Pedi (10)[12], Sotho_Tswana (8)[12]	Xhosa (27)	8,416
SCO Scotland	Late Medieval to post Medieval cemetery at Ensay, Scotland	58	0	62	22	Orcadian (13)[10]	Orcadian (16)	4,739
SOM Somalia	Erigavo District and Darod Kuhar, Somalia	34	0	65	7	Somali (13)[10]	–	6,250
TAN Tanzania	Recent Haya tribe from Lake Victoria, Pare, Gonja, Angoni	43	6	87	19	Hadza (20)[10], Bantu (5)[13]	Pare (23)	3,116
**Total**		**792**	**224**	**1226**	**646**	**1652**	**265**	

Supplementary Table S2. Mean (x̄) and standard deviation (SD) of the dental metric dataset after the removal of individuals with high amounts of missing values, the imputation of missing data, and the transformation into shape variables

		AND	AUS	BEN	ENG	ITA	JAP	JAV	KEN	MEX	MON	NCH	NEG	NGU	PER	PHI	SAF	SCO	SOM	TAN
**UI1-MD**	x̄	1.04	1.05	1.06	1.06	1.06	1.05	1.03	1.05	1.06	1.04	1.05	1.06	1.08	1.04	1.04	1.05	1.07	1.09	1.06
	SD	0.05	0.05	0.05	0.04	0.05	0.04	0.04	0.04	0.03	0.04	0.05	0.05	0.05	0.04	0.04	0.04	0.05	0.04	0.04
**UI2-MD**	x̄	0.85	0.84	0.86	0.84	0.84	0.88	0.85	0.85	0.89	0.87	0.87	0.88	0.86	0.87	0.86	0.85	0.84	0.87	0.87
	SD	0.05	0.05	0.06	0.05	0.03	0.06	0.04	0.04	0.04	0.04	0.04	0.06	0.05	0.04	0.05	0.05	0.06	0.03	0.05
**UC-MD**	x̄	0.95	0.93	0.96	0.95	0.94	0.98	0.96	0.94	0.99	0.95	0.95	0.96	0.95	0.98	0.97	0.96	0.96	0.95	0.95
	SD	0.03	0.04	0.05	0.03	0.03	0.04	0.05	0.04	0.04	0.04	0.04	0.05	0.05	0.03	0.04	0.04	0.04	0.05	0.04
**UP1-MD**	x̄	0.88	0.86	0.87	0.86	0.84	0.92	0.89	0.89	0.91	0.88	0.89	0.90	0.86	0.89	0.90	0.88	0.85	0.90	0.89
	SD	0.03	0.03	0.04	0.03	0.03	0.04	0.04	0.04	0.04	0.03	0.02	0.03	0.03	0.03	0.04	0.04	0.04	0.04	0.04
**UP2-MD**	x̄	0.82	0.82	0.83	0.83	0.83	0.86	0.84	0.85	0.87	0.83	0.84	0.85	0.83	0.86	0.84	0.83	0.83	0.86	0.85
	SD	0.04	0.04	0.05	0.03	0.03	0.04	0.04	0.04	0.04	0.04	0.04	0.04	0.04	0.04	0.05	0.04	0.03	0.04	0.04
**UM1-MD**	x̄	1.29	1.28	1.32	1.32	1.32	1.29	1.30	1.31	1.32	1.28	1.29	1.32	1.33	1.31	1.31	1.32	1.34	1.33	1.31
	SD	0.05	0.04	0.05	0.05	0.03	0.04	0.05	0.05	0.05	0.04	0.03	0.06	0.05	0.05	0.06	0.05	0.05	0.05	0.04
**UM2-MD**	x̄	1.20	1.27	1.24	1.23	1.24	1.24	1.22	1.27	1.27	1.21	1.20	1.22	1.22	1.25	1.22	1.28	1.21	1.24	1.26
	SD	0.07	0.06	0.06	0.05	0.06	0.05	0.05	0.06	0.04	0.05	0.05	0.05	0.06	0.07	0.06	0.06	0.06	0.07	0.06
**LI1-MD**	x̄	0.64	0.65	0.66	0.66	0.65	0.67	0.66	0.64	0.65	0.66	0.66	0.68	0.66	0.66	0.65	0.64	0.68	0.66	0.65
	SD	0.03	0.03	0.03	0.03	0.02	0.03	0.03	0.02	0.03	0.03	0.03	0.03	0.03	0.03	0.03	0.02	0.03	0.03	0.02
**LI2-MD**	x̄	0.72	0.73	0.75	0.74	0.72	0.74	0.73	0.72	0.74	0.74	0.73	0.76	0.74	0.74	0.72	0.73	0.75	0.72	0.72
	SD	0.04	0.04	0.04	0.03	0.02	0.03	0.03	0.03	0.03	0.03	0.03	0.04	0.03	0.03	0.03	0.03	0.03	0.04	0.03
**LC-MD**	x̄	0.84	0.84	0.86	0.84	0.83	0.84	0.85	0.83	0.85	0.84	0.85	0.85	0.84	0.86	0.84	0.86	0.84	0.84	0.85
	SD	0.03	0.03	0.04	0.04	0.04	0.04	0.04	0.02	0.03	0.03	0.03	0.04	0.03	0.03	0.03	0.04	0.04	0.03	0.03
**LP1-MD**	x̄	0.87	0.86	0.86	0.85	0.84	0.90	0.87	0.89	0.86	0.87	0.85	0.88	0.88	0.85	0.87	0.89	0.85	0.88	0.88
	SD	0.04	0.03	0.04	0.04	0.04	0.03	0.04	0.03	0.03	0.03	0.03	0.04	0.04	0.03	0.03	0.04	0.04	0.04	0.04
**LP2-MD**	x̄	0.88	0.87	0.87	0.88	0.88	0.90	0.89	0.91	0.89	0.87	0.86	0.89	0.88	0.89	0.89	0.88	0.87	0.89	0.89
	SD	0.04	0.04	0.03	0.05	0.03	0.04	0.04	0.04	0.04	0.04	0.03	0.05	0.05	0.04	0.05	0.04	0.04	0.03	0.03
**LM1-MD**	x̄	1.38	1.38	1.39	1.40	1.39	1.40	1.40	1.40	1.43	1.40	1.40	1.40	1.42	1.42	1.40	1.39	1.41	1.40	1.39
	SD	0.06	0.05	0.05	0.05	0.05	0.05	0.04	0.05	0.05	0.05	0.04	0.05	0.04	0.05	0.06	0.04	0.05	0.04	0.04
**LM2-MD**	x̄	1.31	1.41	1.31	1.36	1.33	1.33	1.31	1.36	1.36	1.34	1.31	1.30	1.33	1.36	1.31	1.35	1.34	1.32	1.35
	SD	0.05	0.07	0.06	0.06	0.05	0.06	0.06	0.04	0.05	0.05	0.05	0.06	0.07	0.07	0.05	0.08	0.06	0.05	0.05
**UI1-BL**	x̄	0.90	0.89	0.90	0.90	0.90	0.87	0.89	0.89	0.89	0.90	0.90	0.89	0.88	0.90	0.89	0.88	0.91	0.90	0.89
	SD	0.03	0.04	0.04	0.03	0.04	0.06	0.04	0.03	0.03	0.03	0.04	0.06	0.03	0.03	0.04	0.03	0.04	0.03	0.03
**UI2-BL**	x̄	0.82	0.79	0.81	0.80	0.81	0.79	0.81	0.82	0.80	0.83	0.82	0.76	0.79	0.82	0.82	0.81	0.80	0.83	0.81
	SD	0.04	0.04	0.04	0.04	0.04	0.05	0.04	0.04	0.04	0.04	0.04	0.05	0.04	0.04	0.03	0.05	0.05	0.04	0.03
**UC-BL**	x̄	1.01	1.02	1.03	1.03	1.04	0.98	1.01	1.03	1.02	1.02	1.02	0.98	1.02	1.01	1.02	1.03	1.03	1.02	1.02
	SD	0.04	0.05	0.06	0.04	0.04	0.05	0.06	0.05	0.05	0.04	0.04	0.08	0.04	0.05	0.05	0.05	0.05	0.04	0.04
**UP1-BL**	x̄	1.19	1.16	1.15	1.13	1.12	1.18	1.17	1.16	1.13	1.15	1.17	1.19	1.15	1.14	1.18	1.16	1.11	1.15	1.15
	SD	0.04	0.05	0.04	0.05	0.05	0.04	0.05	0.04	0.04	0.04	0.04	0.04	0.05	0.04	0.04	0.05	0.05	0.05	0.05
**UP2-BL**	x̄	1.17	1.16	1.14	1.15	1.16	1.16	1.16	1.15	1.13	1.13	1.15	1.16	1.15	1.13	1.15	1.15	1.14	1.15	1.15
	SD	0.04	0.04	0.06	0.04	0.05	0.05	0.04	0.04	0.06	0.05	0.05	0.05	0.04	0.04	0.05	0.04	0.05	0.05	0.05
**UM1-BL**	x̄	1.45	1.45	1.41	1.43	1.45	1.39	1.43	1.38	1.41	1.41	1.42	1.42	1.44	1.41	1.42	1.39	1.44	1.41	1.39
	SD	0.04	0.05	0.04	0.05	0.05	0.04	0.04	0.04	0.04	0.05	0.05	0.05	0.06	0.04	0.05	0.05	0.04	0.05	0.04
**UM2-BL**	x̄	1.44	1.51	1.41	1.44	1.47	1.39	1.42	1.43	1.41	1.39	1.43	1.40	1.42	1.39	1.43	1.44	1.42	1.42	1.44
	SD	0.06	0.06	0.05	0.05	0.07	0.05	0.05	0.05	0.06	0.05	0.05	0.06	0.04	0.05	0.06	0.05	0.07	0.06	0.06
**LI1-BL**	x̄	0.71	0.71	0.72	0.73	0.75	0.72	0.72	0.70	0.69	0.73	0.70	0.72	0.72	0.70	0.70	0.70	0.74	0.70	0.69
	SD	0.02	0.03	0.03	0.04	0.02	0.04	0.05	0.04	0.02	0.03	0.03	0.06	0.02	0.02	0.03	0.03	0.03	0.02	0.02
**LI2-BL**	x̄	0.77	0.74	0.77	0.78	0.78	0.76	0.75	0.75	0.74	0.78	0.76	0.77	0.77	0.75	0.76	0.75	0.78	0.75	0.74
	SD	0.03	0.03	0.05	0.04	0.04	0.05	0.04	0.04	0.03	0.03	0.03	0.04	0.03	0.02	0.04	0.04	0.04	0.03	0.02
**LC-BL**	x̄	0.94	0.94	0.95	0.95	0.97	0.90	0.95	0.93	0.91	0.95	0.97	0.91	0.93	0.93	0.94	0.95	0.94	0.94	0.94
	SD	0.04	0.05	0.05	0.05	0.05	0.06	0.06	0.04	0.04	0.05	0.05	0.07	0.04	0.04	0.05	0.05	0.05	0.05	0.04
**LP1-BL**	x̄	1.00	1.01	0.98	0.96	0.96	0.98	1.00	1.00	0.95	0.99	1.00	0.99	0.99	0.96	0.99	1.01	0.96	0.99	1.00
	SD	0.05	0.05	0.06	0.04	0.04	0.04	0.05	0.04	0.04	0.04	0.04	0.06	0.04	0.04	0.05	0.05	0.05	0.04	0.05
**LP2-BL**	x̄	1.05	1.03	1.02	1.04	1.04	1.04	1.03	1.03	1.00	1.03	1.02	1.02	1.03	1.01	1.02	1.03	1.03	1.02	1.03
	SD	0.05	0.05	0.05	0.04	0.04	0.05	0.04	0.04	0.03	0.04	0.03	0.06	0.04	0.06	0.05	0.05	0.05	0.05	0.05
**LM1-BL**	x̄	1.34	1.34	1.31	1.32	1.32	1.31	1.32	1.31	1.30	1.34	1.31	1.33	1.31	1.32	1.32	1.30	1.32	1.29	1.30
	SD	0.04	0.05	0.04	0.05	0.03	0.05	0.04	0.04	0.03	0.04	0.04	0.05	0.05	0.04	0.04	0.05	0.04	0.04	0.04
**LM2-BL**	x̄	1.28	1.32	1.26	1.28	1.28	1.28	1.27	1.27	1.25	1.28	1.26	1.25	1.26	1.26	1.25	1.27	1.26	1.25	1.27
	SD	0.04	0.06	0.05	0.04	0.04	0.05	0.05	0.04	0.05	0.04	0.05	0.06	0.04	0.04	0.04	0.06	0.05	0.05	0.05

Supplementary Table S3. Frequencies of positive observations (%) and number of individuals scored (n) of the dental non-metric dataset after the removal of individuals and variables with high amounts of missing values

		AND	AUS	BEN	ENG	ITA	JAP	JAV	KEN	MEX	MON	NCH	NEG	NGU	PER	PHI	SAF	SCO	SOM	TAN
**Premolar accessory cusp (UP1)**	%	6.67	10.2	5.66	2.08	3.17	49.25	22.39	2.82	13.33	37.63	21.43	6.45	20.25	19.15	20.59	7.14	7.14	6.52	6.76
	n	30	49	53	48	63	67	67	71	30	93	28	31	79	47	68	28	56	46	74
**Premolar accessory cusp (UP2)**	%	19.23	39.13	12.77	1.75	6.35	19.4	7.25	2.6	6.9	3.88	14.81	11.11	36.9	4.65	13.64	10	7.27	12	9.72
	n	26	46	47	57	63	67	69	77	29	103	27	27	84	43	66	30	55	50	72
**Carabelli's cusp (UM1)**	%	2.27	6.52	11.84	21.74	26.32	10	16.67	12.35	1.22	4.92	12.5	11.9	14.69	1.27	14.71	19.61	35	23.21	24.39
	n	44	92	76	69	76	70	78	81	82	122	40	42	177	79	102	51	60	56	82
**Hypocone (UM2)**	%	56.1	85.39	65.38	62.67	63.95	87.5	77.78	83.13	56.25	62.83	71.79	66.67	88.55	69.41	80.85	82	64.18	54.84	90.59
	n	41	89	78	75	86	64	81	83	80	113	39	39	166	85	94	50	67	62	85
**Central ridge (LP1)**	%	96.15	94.83	84.62	80.72	67.5	61.76	80	78.57	80	78.87	75	70	97.3	87.88	79.07	62.5	72.73	65	80
	n	26	58	39	83	40	68	65	28	25	71	28	10	37	33	43	24	55	40	50
**Sixth cusp (LM1)**	%	17.65	59.42	4.76	2.86	6.67	42.37	25.93	18.75	30.77	27.5	26.67	18.75	31.52	28.57	36.84	39.29	6.78	10.64	19.15
	n	34	69	42	105	60	59	54	32	65	80	30	32	92	63	57	28	59	47	47
**Seventh cusp (LM1)**	%	9.3	8.79	10.96	5.41	11.43	5.88	8.11	30	2.47	10.2	2.17	8.33	8.7	8.05	9.38	22.86	11.43	26.92	41.94
	n	43	91	73	111	70	51	74	40	81	98	46	36	115	87	64	35	70	52	62
**Deflecting wrinkle (LM1)**	%	24	67.92	13.51	12.87	21.31	20	29.17	52.17	26.92	49.21	47.06	23.33	52.56	50	23.53	40.91	16.33	21.57	41.3
	n	25	53	37	101	61	30	48	23	52	63	17	30	78	42	51	22	49	51	46
**Protostylid (LM1)**	%	8.57	7.59	1.61	7.27	1.52	0	10.17	0	7.04	14.94	3.23	0	0.98	9.09	10.53	0	0	0	1.64
	n	35	79	62	110	66	28	59	37	71	87	31	29	102	66	57	31	59	53	61
**Distal trigonid crest (LM1)**	%	0	2.25	1.41	0.88	1.47	3.39	4.55	0	2.5	2	0	11.43	3.36	1.3	0	2.86	1.43	0	0
	n	44	89	71	113	68	59	66	42	80	100	43	35	119	77	62	35	70	53	65
**Hypoconulid (LM2)**	%	53.85	92.47	37.5	22.55	33.33	80	61.43	74.29	86.21	77.78	78.95	38.24	61.54	85.92	64.62	82.93	26.32	50	83.64
	n	39	93	56	102	60	50	70	35	58	81	38	34	117	71	65	41	57	52	55
Sixth cusp (LM2)	%	5.56	41.43	2.27	0.99	0	9.3	11.11	3.85	11.11	10.14	6.45	6.45	7.07	16.67	12.9	23.33	0	6	19.05
	n	36	70	44	101	58	43	63	26	54	69	31	31	99	48	62	30	53	50	42

Supplementary Table S4. Kinship coefficients (r_{ij}) among 19 population samples based on SNPs

Population	AND	AUS	BEN	ENG	ITA	JAP	JAV	KEN	MEX	MON	NCH	NEG	NGU	PER	PHI	SAF	SCO	SOM	TAN
**AND**	-																		
**AUS**	0.0206	-																	
**BEN**	0.0141	0.0015	-																
**ENG**	-0.0061	-0.0183	0.0259	-															
**ITA**	-0.0059	-0.0202	0.0260	0.0757	-														
**JAP**	0.0216	0.0022	0.0008	-0.0235	-0.0238	-													
**JAV**	0.0217	0.0083	-0.0007	-0.0210	-0.0216	0.0544	-												
**KEN**	-0.0374	-0.0416	-0.0310	-0.0259	-0.0202	-0.0625	-0.0591	-											
**MEX**	0.0043	-0.0077	0.0020	-0.0024	-0.0091	0.0354	0.0192	-0.0593	-										
**MON**	0.0147	-0.0056	-0.0018	-0.0225	-0.0242	0.0628	0.0505	-0.0618	0.0321	-									
**NCH**	0.0152	-0.0014	-0.0015	-0.0205	-0.0226	0.0662	0.0497	-0.0609	0.0428	0.0628	-								
**NEG**	0.0218	0.0236	-0.0001	-0.0242	-0.0232	0.0443	0.0521	-0.0515	0.0140	0.0398	0.0391	-							
**NGU**	0.0218	0.1478	0.0030	-0.0229	-0.0228	0.0022	0.0072	-0.0430	-0.0102	-0.0038	-0.0007	0.0234	-						
**PER**	0.0035	-0.0063	-0.0021	-0.0045	-0.0110	0.0314	0.0177	-0.0574	0.1526	0.0282	0.0328	0.0135	-0.0070	-					
**PHI**	0.0182	0.0051	-0.0028	-0.0249	-0.0254	0.0609	0.0656	-0.0602	0.0226	0.0569	0.0540	0.0601	0.0063	0.0198	-				
**SAF**	-0.0490	-0.0498	-0.0409	-0.0418	-0.0371	-0.0671	-0.0661	0.1229	-0.0617	-0.0701	-0.0674	-0.0578	-0.0482	-0.0619	-0.0667	-			
**SCO**	-0.0062	-0.0164	0.0256	0.0826	0.0728	-0.0243	-0.0203	-0.0284	-0.0042	-0.0224	-0.0217	-0.0245	-0.0219	-0.0054	-0.0251	-0.0449	-		
**SOM**	-0.0305	-0.0368	-0.0116	0.0034	0.0088	-0.0512	-0.0475	0.0640	-0.0428	-0.0484	-0.0484	-0.0422	-0.0367	-0.0485	-0.0499	0.0606	-0.0006	-	
**TAN**	-0.0412	-0.0383	-0.0304	-0.0304	-0.0243	-0.0648	-0.0613	0.1098	-0.0583	-0.0642	-0.0657	-0.0526	-0.0445	-0.0570	-0.0625	0.1294	-0.0329	0.0641	-

Supplementary Table S5. Kinship coefficients (r_{ij}) among 19 population samples based on dental metrics

Population	AND	AUS	BEN	ENG	ITA	JAP	JAV	KEN	MEX	MON	NCH	NEG	NGU	PER	PHI	SAF	SCO	SOM	TAN
**AND**	-																		
**AUS**	0.0184	-																	
**BEN**	-0.0011	-0.0159	-																
**ENG**	-0.0068	0.0231	0.0005	-															
**ITA**	0.0076	0.0385	0.0030	0.1246	-														
**JAP**	-0.0054	-0.0359	-0.0040	-0.0545	-0.1035	-													
**JAV**	0.0182	-0.0164	0.0023	-0.0176	-0.0144	0.0218	-												
**KEN**	-0.0189	-0.0134	-0.0270	-0.0415	-0.0483	-0.0142	-0.0326	-											
**MEX**	-0.0352	-0.0164	-0.0070	-0.0125	-0.0460	0.0316	-0.0069	0.0022	-										
**MON**	0.0189	0.0010	0.0028	-0.0092	-0.0157	0.0206	0.0091	-0.0088	0.0018	-									
**NCH**	0.0074	-0.0159	0.0008	-0.0356	-0.0449	-0.0092	0.0302	-0.0374	0.0030	0.0177	-								
**NEG**	0.0173	-0.0025	0.0218	-0.0305	-0.0597	0.1029	0.0209	-0.0655	0.0000	0.0113	0.0018	-							
**NGU**	-0.0007	-0.0096	0.0061	0.0250	0.0217	-0.0123	-0.0094	-0.0217	-0.0102	-0.0185	-0.0138	0.0085	-						
**PER**	-0.0237	-0.0222	0.0028	-0.0089	-0.0327	0.0200	0.0093	-0.0078	0.0524	0.0172	0.0149	-0.0003	-0.0231	-					
**PHI**	0.0194	-0.0326	-0.0093	-0.0471	-0.0561	0.0149	0.0182	0.0113	0.0100	0.0078	0.0375	-0.0006	-0.0199	0.0116	-				
**SAF**	-0.0156	0.0139	0.0066	-0.0642	-0.0822	-0.0227	-0.0240	0.0722	-0.0011	-0.0298	-0.0206	-0.0307	-0.0074	-0.0154	0.0062	-			
**SCO**	-0.0064	-0.0070	0.0216	0.0951	0.1066	-0.0426	-0.0102	-0.0690	-0.0164	-0.0065	-0.0359	0.0045	0.0382	-0.0126	-0.0507	-0.0656	-		
**SOM**	-0.0247	-0.0581	-0.0076	-0.0089	-0.0079	-0.0373	-0.0133	0.0389	-0.0035	-0.0272	0.0172	-0.0530	0.0092	-0.0086	0.0101	0.0076	-0.0092	-	
**TAN**	-0.0187	-0.0107	-0.0068	-0.0269	-0.0386	-0.0041	-0.0044	0.0380	0.0028	-0.0160	0.0139	-0.0274	-0.0108	0.0003	0.0056	0.0346	-0.0328	0.0361	-

Supplementary Table S6. Kinship coefficients (r_{ij}) among 19 population samples based on dental non-metric traits

Population	AND	AUS	BEN	ENG	ITA	JAP	JAV	KEN	MEX	MON	NCH	NEG	NGU	PER	PHI	SAF	SCO	SOM	TAN
**AND**	-																		
**AUS**	0.0413	-																	
**BEN**	0.0164	-0.0276	-																
**ENG**	0.0102	-0.0926	0.0467	-															
**ITA**	-0.0184	-0.0871	0.0401	0.0871	-														
**JAP**	-0.0220	0.0142	-0.0289	-0.0779	-0.0561	-													
**JAV**	0.0045	0.0020	-0.0108	0.0073	-0.0296	0.0351	-												
**KEN**	-0.0208	-0.0219	-0.0196	-0.0175	0.0101	-0.0647	-0.0430	-											
**MEX**	0.0213	0.0286	-0.0182	-0.0262	-0.0478	0.0423	0.0236	-0.0276	-										
**MON**	0.0114	0.0046	-0.0300	-0.0186	-0.0575	0.0364	0.0414	-0.0023	0.0491	-									
**NCH**	-0.0090	0.0314	-0.0164	-0.0293	-0.0198	0.0523	0.0118	-0.0573	0.0269	0.0174	-								
**NEG**	-0.0012	-0.0137	0.0134	0.0088	0.0175	0.0080	0.0067	-0.0252	0.0022	-0.0220	-0.0099	-							
**NGU**	0.0262	0.0749	0.0073	-0.0488	-0.0250	0.0185	-0.0021	-0.0298	-0.0178	-0.0264	0.0184	0.0111	-						
**PER**	0.0242	0.0393	-0.0263	-0.0354	-0.0670	0.0222	0.0248	0.0103	0.0509	0.0680	0.0093	-0.0173	-0.0094	-					
**PHI**	0.0153	0.0261	-0.0120	-0.0041	-0.0333	0.0325	0.0269	-0.0467	0.0163	0.0264	0.0157	-0.0194	0.0011	0.0186	-				
**SAF**	-0.0405	0.0272	-0.0311	-0.0661	-0.0243	0.0102	-0.0202	0.0443	-0.0062	-0.0200	-0.0085	-0.0003	-0.0173	-0.0046	-0.0129	-			
**SCO**	-0.0212	-0.0806	0.0388	0.0715	0.0865	-0.0323	-0.0189	-0.0156	-0.0518	-0.0532	-0.0124	0.0173	-0.0045	-0.0671	-0.0230	-0.0258	-		
**SOM**	-0.0208	-0.0530	0.0271	0.0305	0.0604	-0.0490	-0.0398	0.0164	-0.0448	-0.0484	-0.0267	-0.0011	-0.0284	-0.0492	-0.0269	0.0198	0.0579	-	
**TAN**	-0.0154	0.0142	-0.0069	-0.0241	-0.0098	-0.0269	-0.0218	0.0560	-0.0287	-0.0155	-0.0262	-0.0256	-0.0030	-0.0041	-0.0090	0.0410	-0.0099	0.0249	-

Supplementary Table S7. Kinship coefficients (r_{ij}) among 13 population samples based on STRs

Population	AUS	BEN	ITA	JAP	KEN	MEX	MON	NCH	NGU	PER	SAF	SCO	TAN
AUS	-												
BEN	0.0021	-											
ITA	-0.0041	0.0028	-										
JAP	0.0001	0.0019	-0.0080	-									
KEN	-0.0118	-0.0101	-0.0112	-0.0142	-								
MEX	-0.0026	0.0014	-0.0049	0.0056	-0.0150	-							
MON	0.0003	0.0022	-0.0067	0.0158	-0.0152	0.0065	-						
NCH	0.0002	0.0021	-0.0076	0.0170	-0.0151	0.0089	0.0166	-					
NGU	0.0146	0.0014	-0.0084	0.0024	-0.0132	-0.0033	-0.0004	0.0008	-				
PER	-0.0023	0.0017	-0.0057	0.0052	-0.0146	0.0426	0.0059	0.0082	-0.0046	-			
SAF	-0.0145	-0.0116	-0.0148	-0.0157	0.0106	-0.0169	-0.0175	-0.0169	-0.0129	-0.0165	-		
SCO	-0.0010	0.0064	0.0105	-0.0048	-0.0095	-0.0022	-0.0034	-0.0041	-0.0054	-0.0015	-0.0127	-	
TAN	-0.0107	-0.0085	-0.0100	-0.0131	0.0145	-0.0149	-0.0147	-0.0150	-0.0105	-0.0149	0.0196	-0.0092	-

Supplementary Table S8. Kinship coefficients (r_{ij}) among 13 population samples based on dental metrics

Population	AUS	BEN	ITA	JAP	KEN	MEX	MON	NCH	NGU	PER	SAF	SCO	TAN
AUS	-												
BEN	-0.0184	-											
ITA	0.0311	0.0016	-										
JAP	-0.0320	0.0026	-0.1079	-									
KEN	-0.0232	-0.0312	-0.0603	-0.0209	-								
MEX	-0.0230	-0.0068	-0.0490	0.0317	-0.0074	-							
MON	-0.0042	0.0046	-0.0192	0.0239	-0.0118	0.0022	-						
NCH	-0.0239	0.0067	-0.0374	-0.0043	-0.0357	0.0051	0.0265	-					
NGU	-0.0115	0.0093	0.0220	-0.0068	-0.0248	-0.0105	-0.0147	-0.0075	-				
PER	-0.0270	0.0037	-0.0325	0.0212	-0.0143	0.0506	0.0177	0.0212	-0.0202	-			
SAF	0.0005	0.0005	-0.0942	-0.0279	0.0566	-0.0107	-0.0345	-0.0238	-0.0113	-0.0237	-		
SCO	-0.0103	0.0247	0.1151	-0.0344	-0.0728	-0.0149	-0.0055	-0.0315	0.0409	-0.0113	-0.0686	-	
TAN	-0.0167	-0.0073	-0.0408	-0.0072	0.0328	-0.0023	-0.0163	0.0189	-0.0109	-0.0017	0.0284	-0.0333	-

Supplementary Table S9. Kinship coefficients (r_{ij}) among 13 population samples based on dental non-metric traits

Population	AUS	BEN	ITA	JAP	KEN	MEX	MON	NCH	NGU	PER	SAF	SCO	TAN
AUS	-												
BEN	-0.0354	-											
ITA	-0.0998	0.0710	-										
JAP	0.0076	-0.0326	-0.0548	-									
KEN	-0.0396	-0.0152	0.0045	-0.0766	-								
MEX	0.0270	-0.0108	-0.0397	0.0310	-0.0314	-							
MON	0.0057	-0.0291	-0.0581	0.0309	-0.0049	0.0445	-						
NCH	0.0234	-0.0093	-0.0089	0.0452	-0.0730	0.0217	0.0137	-					
NGU	0.0722	0.0041	-0.0259	0.0134	-0.0426	-0.0169	-0.0262	0.0177	-				
PER	0.0380	-0.0282	-0.0721	0.0135	0.0070	0.0472	0.0666	0.0013	-0.0120	-			
SAF	0.0095	-0.0316	-0.0303	0.0038	0.0270	-0.0114	-0.0177	-0.0239	-0.0257	-0.0069	-		
SCO	-0.0918	0.0635	0.1235	-0.0301	-0.0166	-0.0453	-0.0529	-0.0012	-0.0054	-0.0716	-0.0275	-	
TAN	0.0022	-0.0086	-0.0124	-0.0318	0.0475	-0.0292	-0.0128	-0.0336	-0.0125	-0.0045	0.0313	-0.0116	-

References

1. Hanihara, T. & Ishida, H. Metric dental variation of major human populations. *Am. J. Phys. Anthropol.* **128,** 287–298 (2005).
2. Hanihara, T. Morphological variation of major human populations based on nonmetric dental traits. *Am. J. Phys. Anthropol.* **136,** 169–182 (2008).
3. Pemberton, T. J., DeGiorgio, M. & Rosenberg, N. A. Population structure in a comprehensive genomic data set on human microsatellite variation. *G3 (Bethesda)* **3,** 891–907 (2013).
4. Reich, D., Thangaraj, K., Patterson, N., Price, A. L. & Singh, L. Reconstructing Indian population history. *Nature* **461,** 489–494 (2009).
5. Pugach, I., Delfin, F., Gunnarsdottir, E., Kayser, M. & Stoneking, M. Genome-wide data substantiate Holocene gene flow from India to Australia. *Proc. Natl. Acad. Sci.* **110,** 1803–1808 (2013).
6. Qin, P. & Stoneking, M. Denisovan Ancestry in East Eurasian and Native American Populations. *Mol. Biol. Evol.* **32,** 2665–2674 (2015).
7. Abdulla, M. A. *et al.* Mapping human genetic diversity in Asia. *Science* **326,** 1541–1545 (2009).
8. Nelson, M. R. *et al.* The Population Reference Sample, POPRES: a resource for population, disease, and pharmacological genetics research. *Am. J. Hum. Genet.* **83,** 347–358 (2008).
9. Altshuler, D. M. *et al.* Integrating common and rare genetic variation in diverse human populations. *Nature* **467,** 52–58 (2010).
10. Lazaridis, I. *et al.* Ancient human genomes suggest three ancestral populations for present-day Europeans. *Nature* **513,** 409–413 (2014).
11. Xing, J. *et al.* Toward a more uniform sampling of human genetic diversity. A survey of worldwide populations by high-density genotyping. *Genomics* **96,** 199–210 (2010).
12. Xing, J. *et al.* Fine-scaled human genetic structure revealed by SNP microarrays. *Genome. Res.* **19,** 815–825 (2009).
13. Lopez Herraez, D. *et al.* Genetic variation and recent positive selection in worldwide human populations: evidence from nearly 1 million SNPs. *PLoS One* **4,** e7888 (2009).
